# RE-Europe, a large-scale dataset for modeling a highly renewable European electricity system

**DOI:** 10.1038/sdata.2017.175

**Published:** 2017-11-28

**Authors:** Tue V. Jensen, Pierre Pinson

**Affiliations:** 1 Technical University of Denmark, Department of Electrical Engineering, Elektrovej 325, 2800 Kgs Lyngby, Denmark

**Keywords:** Renewable energy, Energy supply and demand

## Abstract

Future highly renewable energy systems will couple to complex weather and climate dynamics. This coupling is generally not captured in detail by the open models developed in the power and energy system communities, where such open models exist. To enable modeling such a future energy system, we describe a dedicated large-scale dataset for a renewable electric power system. The dataset combines a transmission network model, as well as information for generation and demand. Generation includes conventional generators with their technical and economic characteristics, as well as weather-driven forecasts and corresponding realizations for renewable energy generation for a period of 3 years. These may be scaled according to the envisioned degrees of renewable penetration in a future European energy system. The spatial coverage, completeness and resolution of this dataset, open the door to the evaluation, scaling analysis and replicability check of a wealth of proposals in, e.g., market design, network actor coordination and forecasting of renewable power generation.

## Background & Summary

Most countries around the world have high ambitions in terms of deployment and integration of renewable energy generation capacities^1–3^. Such a rapid increase in renewable energy penetration comes with technical, economical and regulatory challenges. Power systems and electricity markets were originally designed for conventional (say, thermal, nuclear and hydro-based) and centralized power generation, while near-future systems are set to rely on distributed and non-dispatchable generation as triggered by the deployment of renewable power generation capacities. These produce energy with nearly-zero marginal cost according to Nature’s will, with the inherent characteristics of being highly variable and of limited predictability. This induces substantial challenges in the operation of power systems and electricity markets^4^.

In that context, decision problems in operation and planning of power systems ought to rely on large-scale models and datasets allowing us to weigh and appraise alternative options in an ever more dynamic and uncertain environment. Assembling such models is often made difficult by a combination of confidentiality issues and competitive industrial interests. This is complemented by the need to take a multidisciplinary approach since the renewable energy system combines aspects of meteorology, economics and power systems engineering. It is our objective here to make a first proposal for such a large-scale dataset for the whole Europe in an open-access format for all researchers to have a common playground to benchmark their own ideas, scale them up to a large system, but also replicate and build on those of others. The resulting Renewable Energy Europe (RE-Europe) dataset allows envisaging a future where a large part of the energy consumption may be met by renewable energy generation. It may then readily be used for assessing market designs over the whole Europe, e.g., flow-based coupling of day-ahead markets^5,6^, benchmarking new cross-zonal coordination mechanisms for ancillary services^7,8^ or comparison of alternative forecasting models^9,10^. We see a multitude of other applications for the dataset in the fields of, e.g., energy systems, statistics, complex networks, economics and mechanism design, comprising too many to be extensively listed here.

Previous efforts in building open system models have focused on specific sections of such a model, e.g., the transmission system and generation capacity^11–15^ or the renewable infeed^16,17^, provide some data only at an aggregate level^18^, or cover only a portion of Europe at high detail^19^. In addition, open tools have been developed which allow putting together portions such a data set^13,16,20^, for overviews, see^21,22^. Building on these previous efforts, we provide a complete, ready-to-use, open license dataset of the full electricity system at a high level of detail.

**Figure 1:**
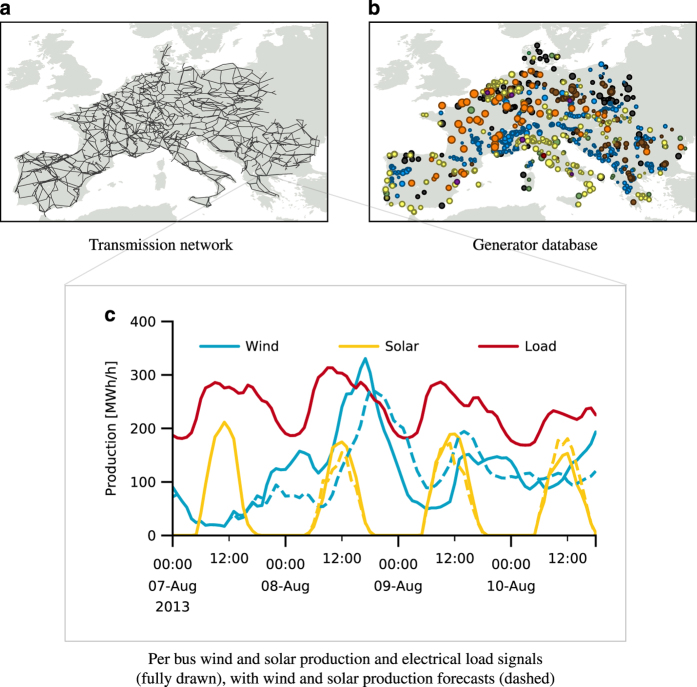
Overview of dataset components. (**a**) Transmission network, (**b**) Generator database, (**c**) Per bus wind and solar production and electrical load signals (fully drawn), with wind and solar production forecasts (dashed).

Assembling the RE-Europe dataset required defining a number of individual components, which comprise the separate, but coupled components of the data set. These include the power network, conventional generators, as well as renewable energy generation and electric power demand. While the first two may be described in a static manner, in terms of their technical and economic characteristics, weather- and demand-related signals are best described in a dynamic manner with sequences of forecasts and observations in the form of time-series. These components are outlined in Fig. 1. Since most renewable generating capacities being deployed today are for wind and solar power, we will concentrate on these here^23^.

The electric power system is a physical network, connecting people and industries with intricate demand patterns, and now, distributed renewable energy generation with complex spatial and temporal dependencies. As all electrical interactions on the network happens through injection or extraction of energy through the nodes of this network, renewable production and demand signals are to be aggregated at the node level. In practice, then, the physical network model defines the granularity of the dataset. The granularity is key here, as considering, for instance, a single node per country would most likely mask some of the important effects to look at in a renewable energy future (e.g., bottlenecks in transporting power between Northern and Southern parts of Germany), while using a fully detailed grid model increases computational burden disproportionately. Attempts at proposing sensible open-access network descriptions already exist^11–14^ and we build on those here.

When it comes to conventional generators in Europe, one naturally cannot get access to their full detailed information (both technical and economic) in view of industrial interests. Initiatives for summarizing their most important characteristics for operations and planning studies already exist, as an example in the GlobalEnergyObservatory database^15^ that was used as a basis here. This database contains location, fuel type and other basic characteristics of these generators, covering in total 78% of European generation capacity. On top of that, we complement the unit descriptions with some important characteristics for more complete studies, including cost data and minimum online capacity using standard methods from the power market literature. Full details are given in the Methods section.

While renewable power production signals and forecasts at the level of each node are generally not available due to commercial interests, even if they were, such signals only serve to describe the production capacity in place today. Since a large part of the renewable energy infrastructure has yet to be built, the production signals of today only inform in a limited manner about the impact of future installations. To describe the potential wind and solar power available at each node, we use the method of Andresen *et al*.^16^ to convert high-resolution numerical weather data using simple physical models of wind turbine and solar panel power curves. This approach represents wind and solar spatio-temporal production patterns, while allowing modeling renewable production capacity even where none exists today. By using forecasts from numerical weather systems, our production forecasts capture the full spatio-temporal dynamics of the underlying meteorological fields.

Eventually, we obtain a large-scale dataset that has the fundamental information required to study a renewable energy future in Europe, as a basis for operational and planning studies. Each component can be individually extended in the future. An overview of each component is given on Fig. 1, with the size and resolution of each given in Table 1. For a detailed discussion of each component, see the relevant sections below. Foreseen improvements to the current dataset include longer sequences for the dynamic information (permitting to simulate a wider range of operating conditions), probabilistic information on renewable power production and power consumption and extending the network to cover the remaining areas of Europe.

## Methods

The dataset consists of three static components (network, generators and installed renewable capacity per node),and three dynamic components (demand signal, renewable production signals and renewable production forecasts). The following paragraphs detail how these components were derived from their primary sources, the challenges inherent in their construction, as well as the value of their inclusion within the whole dataset. Further, we discuss the potential and ease of extension of each component.

### Network georeferencing

The electricity grid acts as the connector of all resources, its size and resolution determining which phenomena may be examined. As this data set aims at resolving and representing effects stemming from renewable energy production, the network should be large and detailed enough to capture and resolve the dynamics of available renewable resources. Such dynamics are closely related to the size and resolution of weather systems, which may stretch for thousands of kilometers and have dynamics which, when averaged over the hour, are smaller than ~50 km, as expressed through the synoptic scale speed, typically 7–10 m/s (ref. 24). To accommodate both the extent and resolution of renewables, the most pertinent network model available is that of Hutcheon and Bialek^11^, which comprises a network of 1,494 buses connected by 2,322 conductors which form 2,156 transmission lines, with flow limits on cross-border lines. This network model covers mainland Europe, excluding the British Isles, Fennoscandia and the Baltic states, and is thus extensive enough to cover several weather patterns. Further, the model covers transmission lines down to the 110/220 kV voltage level, with a typical transmission line length of 30–70 km. Both the extent and resolution of the grid thus make it well-suited to model the hourly-averaged renewable infeed. While other extensive and detailed grid models exist, e.g.,^12,13,19^, these either cover a smaller area than^11^, or are not easily georeferenced. Due to the modular structure of the RE-Europe data set, future versions can switch to these alternate grid models as and when it is deemed advantageous to do so.

While this network model is extensive, and provides an accurate network layout, it does not contain the latitude and longitude of buses. The geographical locations of the buses are important as they define which resources, e.g., generators and wind production capacity, are attached to which buses. For our purposes, this information is critical to use the grid model, and the geographical coordinates of the buses must be reconstructed.

The network comes with a set of display coordinates $\vec{y}$ which correspond to each bus’ map coordinates on the ENTSO-E Grid Map. In order to extract these, however, the proprietary program in which the network is delivered requires using another coordinate transformation. Thus, the coordinates available after extraction $\vec{z}$, are related to the actual latitudes and longitudes of each bus $\vec{x}$ as shown schematically below

(1)x→ENTSO‐EGridMapProjection→q→Workby[11]→y→ExtractionfromProgram→z→

From the length of this chain, recovering $\vec{x}$ by inverting map projections is clearly not a feasible strategy. Instead, we seek to fit a smooth function *f* which takes the extracted coordinates $\vec{z}$ for each bus and outputs the geographical location $\vec{x}$ of that bus.

To this end, we fit *z*_*n*_ for a subset of buses to the geographical coordinates *x*_*n*_ of their associated towns, and use the fit to approximate $\vec{x}$ for the remaining buses. Provided the fit achieves errors of less than ~50 km, the renewable energy signal should not be adversely affected Due to the entry process for the underlying grid, where parts of the grid were entered at a time and then stitched together, small inconsistencies exist between bus positions along country borders. Selecting a very high number of buses is likely to be adversely affected by these inconsistencies, degrading the quality of the fit.

We find that using a third-order polynomial ansatz for *f* along with 34 buses, chosen for their geographical coverage, yields a fit with a small error while avoiding overfitting. The fit was carried out by ordinary least-squares. For details on the outcome and the buses chosen, see the Technical Validation section.

### Generator data

As Europe has no official database of generators, we use the curated open-access generator database^15^. From the database, each generator’s geographical coordinates, production capacity and fuel type is listed. The production capacity coverage for the relevant part of Europe is 78%, with the lowest individual coverages being Switzerland (53%), Austria (54%) and Slovenia (62%), as compared to 2012 numbers^25^.

The database does not list economic parameters for the generators, as these are typically not publicly available. In view of the confidentiality issues, and that we would not want to discriminate among power plants at such large scale, we choose to estimate the economic parameters for each plant based on their fuel types. The choices made below are to be seen as a representative frame in which to study future highly renewable energy systems. That is, they should neither be seen as a scenario, nor as an optimized portfolio, for a future generation mix.

The marginal costs of each fuel type is based on the data in ref. 26, which gives variable operation and management cost (O&M) estimates including fuel cost for new plants erected in 2019. We note, that CO_2_ prices are not included in the marginal price estimates. A wealth of other sources of cost data exist, e.g.,^23,27,28^. Cost estimates given in these sources span a wide range, e.g.,^28^ gives a range of 50–100 DKK2013/GJ for gas prices in 2020, leading to a comparable spread for the marginal cost of electricity from gas turbines at this time. In the interest of providing a complete dataset, we choose to limit ourselves to the median numbers given in ref. 26.

To comply with the limitations of ref. 26, the 67 fuel types present in the generator database^15^ are reduced to 9 generic types. As only 5 fuel types are described in ref. 26, covering the other 4 fuel types requires additional assumptions. To assign specific numbers to each fuel type, we make the following assumptions about plant types and production cycles: Coal, lignite, oil, and natural gas plants are assumed to be outfitted with carbon capture and sequestration systems. Coal and lignite are assumed to use the efficient but experimental integrated gasification and combined cycle (IGCC) process for production, while oil and gas use the combined cycle. Plants with unknown fuel type are taken to have the highest marginal cost of all technologies. For the fuel types not listed in ref. 26, we assume that the non-fuel portion of the O&R cost is the same for similar plant types, and that only the fuel cost changes. As lignite is nearly interchangeable with coal in operation^29^, and oil plants can run on the same cycle types as the gas fired plants^30^, we use costs for coal plants to calculate costs for lignite plants, and costs for gas plants to calculate costs for oil plants.

**Figure 2:**
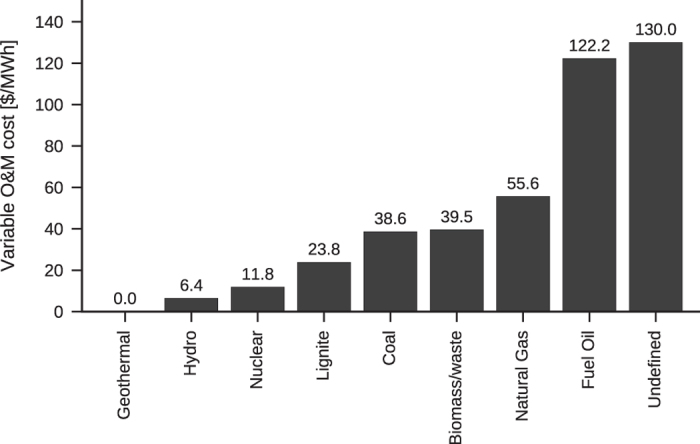
Variable operational & marginal (O&M) costs per fuel type.

To compute the fuel-only costs, the following sources are used. Price of coal from NYMEX QL^31^, and the price gas are the Henry Hub natural gas index price^32^, both from the 8th of August 2014. The price of fuel oil is the Rotterdam IFO 380 index price on the 7th of August 2014 (ref. 33). Lignite has no exchange price, as it is mostly used in the local area of the mine, but its price is historically about a third of the coal price^34^. Heat values are from ref. 35, and thermal efficiencies are from ref. 30. Both of these parameters for lignite are assumed the same for lignite as for coal. Table 2 gives the estimates of fuel cost used to compute variable O&M for lignite and fuel oil, with the final variable O&M costs given on Fig. 2. The above price for oil is no longer representative, as the price of fuel oil has been cut in half since the dataset was compiled^36^. Due to the volatility of fuel prices, these costs should be taken as representative, and we encourage users to provide their own fuel cost estimates in accordance with their use case.

If all plants were to be set to exactly the same marginal cost, the system would have a high degree of degeneracy, which can lead to computational problems when optimizing for the lowest-cost generator dispatch. In reality there is some variation in costs between different plants, owing to geographical variation in prices and technological variance. As an example, the levelised costs of electricity are found in ref. 26 to vary by approximately 10% due to these factors. Given the scale of our dataset, and the lack of available industrial expert knowledge, one cannot retrieve real values for these variations. Without relevant specific knowledge, the best way to achieve required diversity of costs figures is by incorporating a random component. To model cost variation in a simple way, we multiply the marginal cost of each plant by a factor chosen uniformly at random from 90 to 110%. For any specific use case that may be sensitive to localized information on variation in marginal costs of electricity, this could be examined e.g., by Monte Carlo simulation.

In order to model unit commitment problems, additional generator parameters are needed. With the wealth of possible cycle types, number of valves and steam engines, variations in boiler, turbine, and generator designs, and consideration of the ages of the plants, it is impossible, based on publicly available data, to accurately describe these parameters for each plant type. Instead, our goal is the more modest one of giving reasonably representative values for each fuel class of the plants. It is our expectation, that any research which require more nuanced estimates will provide these themselves.

Table 3 gives the chosen parameters for unit commitment problems, with ranges indicated in parens. The parameters were selected as follows: When a plant shuts down, and is subsequently started back up, additional fuel is used to bring the plant fully online, and thermal stresses may cause wear on components. The cost of this is called the cycling cost of the plant. Kumar *et al*.^37^ list cycling costs for coal and gas plants depending on the cycle type of the plant. For both fuel types, the median value for warm start is used, with the coal and gas unit types taken to be large sub critical and combined cycle gas turbine (CCGT), respectively. Lignite-fired, biomass/waste, geothermal, and plants with unknown fuel type are assumed to have the same cycling costs as coal-fired plants due to their similar cycle types, while fuel oil-fired plants are giving the same cost as natural gas-fired ones. As reported by Nilsson and Sjelvgren^38^, the start-up costs of hydro vary by about a factor of three, depending on the operator and plant type. From their Fig. 2, a middling estimate of 150$ for startup of a 50MW turbine is found, corresponding to a startup cost of 4.3$/MW in 2012 dollars. No good source of nuclear plant cycling costs could be found. Usually, these plants are run as baseload plants, and are not taken offline except for maintenance. To reflect this, they have been assigned a very high cycle cost compared to the other plants.

When a plant is delivering power, it has a minimal amount of power that can be delivered during stable operation, called the minimal online capacity. For gas turbines^39^, lists 40% as a state-of-the art figure. This figure is also assumed to hold for the fuel oil plants, and for plants with unknown fuel type. Coal plant parameters are found in ref. 40, and lignite plants are assumed to be equal to these. The minimal online capacity of a hydro turbine depends on the type of turbine, but all types lose efficiency at or below 15% of rated power^41^. Though this limit may be circumvented by redirecting water between multiple turbines, we use 15% as an estimate of the total plant minimal production capability. For nuclear plants^42^, states that European regulations do not require a lower online capacity than 50%, but that manufacturers set their own standards, typically 20%. We use the latter number as our estimate. No source could be found for the minimal online capacity of a geothermal plant, and we arbitrarily set it to 15%.

Unit commitment models often include constraints which limit the amount to which plants can be turned on and off. Typically, these specify that if the plant is turned off, it cannot be restarted before a certain amount of time, called the minimum down-time. Similarly, the plant cannot be shut down after starting up before the minimal up-time has passed. However, there is a dearth of data on what these times are for different plants, and discussion if such times are able to capture the physical operation of plants^43^. A commonly used source for up- and down times is Grigg *et al*.^44^, but no sources are listed for the numbers given. Furthermore, the up- and downtimes in this source depend more on the size of the respective plants rather than their fuel type or cycle. This indicates that grouping plants by fuel as has been done thus far is insufficient to capture model up- and downtimes. To provide a complete data set, we report the values in Table 3, which are consistent with mid-sized plants from Grigg *et al*.^44^.

### Definition of demand signal

The electrical demand signal, or load signal, informs the need for system balancing and use of resources. In particular, the spatial distribution of the load signal defines the need for transmission, as power may be generated far from consumption centers. From the ENTSO-E website^45^, data for the aggregated demand of each country is available hour by hour. However, the current application requires data at the resolution of the network model. This makes it necessary to disaggregate the load signal, i.e., to split the country-aggregate load signal into its many component signals.

This disaggregation is an impossible task to achieve exactly, and we instead content ourselves with a heuristic approach. Intuitively, it is reasonable to project according to demographic or economic data; the more people live in an area and the more industry is in that area, the higher we would expect the electricity demand of that area to be. This notion is supported by^46^, who observe that the load in regions of Italy is reasonably correlated with the population in that zone. In contrast^47^, finds power consumption to be slightly negatively correlated with relative regional population, but strongly correlated with relative regional GDP.Given the lack of detailed maps of GDP, as compared to the relative ease of obtaining highly detailed population data, we choose to project the demand signal proportional to the population.

For this purpose we use the population density data of^48^, sampled at the center of each grid cell of a coordinate grid consistent with the ECMWF renewable energy data (grid resolution 0.25°×0.25°). This population density is multiplied by the area of the grid cell to obtain an estimate of the population in the grid cell. For each country, the aggregate consumption time series is then distributed on the onshore grid cells for that country proportional to estimated population. The end result is a map of mainland Europe with each country having a spatially static demand pattern which fluctuates in time according to the country-aggregate data.

The resulting synthetic demand data describes the effect of national load centres on flows, but is naturally unable to describe dynamic intra-country variations. However, extending the demand signal to include such dynamic inhomogeneities is currently impossible given the lack of open-access sources of demand at higher spatial resolution.

### Renewable energy signals and forecasts

As wind- and solar-driven energy sources come to dominate energy production, system operation is critically dependent on weather patterns. Where and when situations of high and low solar and wind availability occur inform the need for use of the transmission system and/or activation of dispatchable generation. Mis-estimation of the need for and use of these resources may thus result from failure to accurately model the spatio-temporal structures of the weather. To properly represent these structures, we utilize data from numerical weather prediction systems. These use data from a multitude of weather monitoring stations and related sources to estimate the past, present and future state of the atmosphere, storing the output as meteorological field data. Such data has previously been used to obtain approximate renewable energy production signals, see Andresen *et al.*^16^ for an overview.

When constructing wind- and solar production signals by the method described here, the outcome is necessarily synthetic, and will not reproduce real-world signals exactly. A proper term for the signals produced would be ‘synthetic observations and forecasts’, possibly with an additional moniker relating that the signals capture the aggregate space-time dynamics of the real-world weather signal. Still, since actual bus-level production data is not typically shared, and in the interest of brevity, we shall refer to the signals as simply ‘observations’ and ‘forecasts’ below.

Based in this line of work^49^, develops a framework for wind and solar energy conversion, which forms the basis for the present data set. In this framework, wind speed and insolation fields are converted into production factors through wind turbine and solar panel models. The wind turbine model converts wind speed into produced power using a power curve. Based on the recommendations in ref. 49, we use a smoothed power curve for a Siemens SWT 107 turbine for both onshore and offshore wind. This turbine is suggested as a good proxy for future turbine fleets, for which the majority of production will come from very large units. For the solar panel, we use the Scheuten P6–54 215 Multisol Integra Gold^50^. This panel is of a typical size for residential installations. For simplicity, we assume all panels are mounted at a 30 degree angle from zenith and facing due south. A more realistic conversion would angle the panels optimally, and use a mixture of orientations to represent the diversity of conditions under which these panels are installed. We leave these considerations on the best mix of panel parameters and turbine types to be addressed in a future version of the dataset.

As input to the conversion, we use the ECMWF deterministic forecast^51^ and the COSMO-REA6 data set^52^ for the years 2012 to 2014. The ECMWF deterministic forecast is provided every 12 h for the subsequent 90 h at hourly time steps, with a spatial resolution of approximately 16×16 km. The COSMO-REA6 data set contains hourly data at a spatial resolution of approximately 7×7 km. In order to use these data set in the conversion process, additional processing is needed.

For conversion to wind turbine power factors, the wind speed at turbine hub height (80 m) is needed. However, neither data set supplies wind speeds at this height, and an interpolation method must be used. In the ECMWF data set, wind speeds are given at 10 and 100 m. For such data^53^, recommends using logarithmic interpolation in this case, which for wind speeds *v*_1_ and *v*_2_ at heights *h*_1_ and *h*_2_ yields an expression for the wind speed at height *h*. In contrast, the COSMO-REA6 data set gives values of the wind speed at 6 different layers of the atmosphere from a near ground level to ~240 m above the ground. For the COSMO-REA6 data, we follow the advice of^53^, and use linear interpolation between model levels to obtain the wind speed at turbine hub height.

For solar power conversion, the beam (or direct), diffuse and ground-reflected components of insolation at short wavelength are needed. All three components are directly available from the COSMO-REA6 data set, while the ECMWF data set only provides the sum of the beam and diffuse fields. To determine the portion of incoming insolation from the diffuse component for the ECMWF data, the method of Reindl *et al.*^54^ is used. We omit a complete description of the solar conversion method for brevity, and refer the interested reader to^49^ for a more detailed description.

### Adapting wind and solar forecasts to form a continuous signal

The forecasts issued in the ECMWF data set may be inconsistent with the realized production as given by converted data based on COSMO-REA6 data set. For modeling purposes, it may be beneficial to have a continuous signal that is consistent with the forecast signal. Consequently, we use the ECMWF forecasts as a basis to produce another continuous signal, to be seen as an alternative realization. This new signal is referred to as the ECMWF signal, not to be confused with the ECMWF forecast or the COSMO signal.

The inclusion of continuous signals derived from both ECMWF and COSMO-REA6 data means the data set comes with two ‘truths’, one derived from the ECMWF data, and one from the COSMO-REA6 data. Naturally, neither of these signals conform exactly to real-world production, due to errors inherent in the type of modeling used here, or to each other, owing to differences in the derivation of the meteorological fields and conversion procedures. By including two signals, our dataset offers a built-in check to which extent results are sensitive to the specific renewable energy signal used.

We form a continuous signal from the ECMWF data by stitching together forecasts as they become available: A new forecast is available every 12 h, so we take the first 12 h of each forecast and stitch them together to form the signal. As the ensemble mean forecast represents a best guess of the actual physical trajectory of the system, it is reasonable to expect the synthetic signal to track the real world. However, due to inherent inaccuracies and uncertainty in numerical weather prediction, there will be differences in wind speeds across the seam between two forecasts. This unphysical jump is a concern in examining systems which are coupled in time. For the power system application considered here, it represents large, recurring ramp events. To avoid the impact of these ramp events, the seam must be smoothed over in a way which does not adversely affect the spatial information in the wind time series. To the best of the authors’ knowledge, this problem is unexplored in the climate reanalysis literature.

As there need not be a physical path connecting the forecasted state some time steps before the seam to the state some time steps after the seam, a detailed physically-motivated model is not guaranteed to be able to solve the issue of joining forecasts. As such, we are not looking to construct a physical system trajectory, but rather to smooth out the seam in a sensible way through interpolation. The important characteristics that we wish to keep in the wind data are the typical ramp characteristics of the local wind field, and the spatial patterns of wind production. The spatial patterns are approximately preserved in the Empirical Orthogonal Function (EOF) decomposition of the wind fields^55^, and we base our interpolation method on this.

To construct the EOF components, let ${\vec{w}}_{t}$ be a vector of the u- and v components of the wind field at the *N* positions of the grid,

(2)w→tT=(ux1,t,…,uxN,t,vx1,t,…,vxN,t),

and construct the correlation matrix *C* of the wind signal as the outer product of these *w*’s, averaged over time

(3)C=〈w→tw→tT〉t=1TWWT.

Let ${\vec{µ}}_{1},\ldots ,{\vec{µ}}_{N}$ be the orthonormal eigenvectors of *C*, and construct the eigensignals of ${\vec{w}}_{t}$ as

(4)ki(t)=µ→i⋅w→t.

Using an interpolation method to construct the interpolated eigensignal ${\tilde{k}}_{i}(t)$, the interpolated wind signal is found as

(5)w˜→t=∑ik˜i(t)µ→i.

Since the interpolation is done on the eigensignals, the spatial characteristics of the wind signal are preserved. The use of a proper interpolation method will ensure that the temporal characteristics are not unduly disturbed.

The method as described above is computationally very expensive, as *N* is very large, and the task of finding eigenvectors scales poorly with *N*. In order to make the problem computationally feasible, we first divide the signal into months, and compute a correlation matrix *C*_*m*_ for each month separately. We then exploit that since the number of hours in a month ≪*N*, the matrix *C*_*m*_ is of much lower rank than *N*. This means, that we can instead find the eigenvectors *ν*_*i*_ of *W*^*T*^*W*, and re-construct *μ*_*i*_ via

(6)µi=WTνi,

with appropriate normalization. The use of a monthly correlation matrix restricts the dimensionality of the span of *μ*_*i*_, but as we are merely seeking a reasonable method to smooth out the signal, this is not a great concern. In performing the actual interpolation of *k*_*i*_(*t*), a cubic spline method is used across each seam. For the results of this interpolation, see the discussion in the Technical Validation section.

For solar data, the answer to the problem of a seam is much simpler; The first 24 h of the forecasts issued at midnight are used instead of the first 12 h from every forecast. Apart from some of the northernmost areas of Norway, there is never any solar radiation across the seam, and the problem is avoided altogether. This does mean that a user of the dataset should take care if the forecast issued at midnight is used to schedule production for the period from noon to midnight of the following day, as the solar production forecast will be perfect at this time; see the Technical Validation section.

### Aggregation of renewable energy and load signals to nodes

The previous sections define maps of renewable energy production and demand on a series of grid cells. To combine these maps with the network data requires aggregating onto the space of nodes. For this purpose, it is reasonable to assume that a renewable installation or consumption area will be connected to the closest transmission substation, as this leads to the lowest cost of setting up the connection. This implies that each node aggregates consumption and production from its nearest grid cells. Our aim in this section is to construct matrices which project a vector of data defined for each grid cell onto the space of nodes in such a way that each node receives data from the area closest to the node.

For aggregating the solar signal, we consider areas available for PV installation if they are onshore. For aggregating the wind signal, we consider both onshore and offshore grid cells available for installation, with offshore grid cells only available provided the water depth is less than 70 m. This latter depth is a conservative estimate of the maximum water depth for wind turbine installation for near-future technologies, see^56^. Through this process, offshore wind is thus mixed in with onshore wind, such that nodes with a significant share of offshore grid cells will see their signals dominated by offshore wind. To rectify this, we intend to provide separate time series for off- and onshore wind in future versions. Last, when aggregating the demand signal, all grid belonging to the onshore areas of countries covered are aggregated, even if those grid cells belong to areas which are not covered by the grid, e.g., eastern Denmark and Corsica.

When aggregating load data, the total load across Europe should be preserved. For this purpose, a projection matrix that preserves the sum is required, call it *M*^*SP*^. On the other hand, when aggregating capacity factors for wind and solar, a projection matrix that averages over the underlying area is required, call it *M*^*AP*^. Each of these matrices should define a proportional sharing rule between each grid cell and its associated nodes. A node and a grid cell are considered associated if either (1) the node is closer to the grid cell than any other node or (2) the grid cell is closer to the node than any other grid cell. This ensures that each node receives data from at least one grid cell. Further, if a grid cell is associated to multiple nodes, the contribution from that grid cell should be split evenly among all associated nodes.

The construction of *M*^*SP*^ and *M*^*AP*^ proceeds as follows: Let *P* be the set of grid cells in the map, P′⊂P the region over which we wish to aggregate the data, and *N* the set of nodes in the graph. Let $M\in R^{\left|N|\times |P\right|}$ be a matrix initialized with all zeros. Then, for all nodes *n*∈*N* and grid cells p∈P′, set *M*_*np*_=1 if (1) *n* is closest to *p* or (2) *p* is closest to *n*.

Using *M*, we find *M*^*SP*^ and *M*^*AP*^ as

(7)MnpSP=Mnp∑n′∈NMn′p

(8)MnpAP=MnpSP∑p′∈P′Mnp′SP.

These matrices aggregate load data maps and maps of renewable energy capacity from grid cells to each node.

In the construction of *M*^*AP*^, we implicitly assume that the weight given to each grid cell is equal. However, some grid cells cover a larger area than others, such that their contribution to the average should be greater. We choose to avoid this complication, as this difference over the domain covered by the network is less than 5%.

### Definition of renewable energy capacity layouts

Following aggregation, the load data is in units of MWh, while the renewable production signals are given as the estimated production relative to installed capacity, which at this point is undefined. Though a layout representing the capacity installed in current systems may be of interest, the lack of good open-source databases for solar installation in particular makes such layouts impossible to generate. Further, as the bulk of renewable energy installations have yet to be built, the current-day layout of capacity does little to inform on future energy systems.

To provide a ready-to-use data set, we define two capacity layouts, which we term the *uniform* and the *proportional* layouts. In the uniform layout, installed capacity is spread uniformly across Europe, and the capacity of each node will be proportional to the area aggregated by the node. In the proportional layout more capacity is installed in areas with high renewable production potential, such that the capacity for an area is proportional to the yearly mean capacity factor for that area.

The uniform and proportional layouts lack a constant factor to set the total installed capacity. We fix this constant factor, such that using the capacity layout directly represents a situation of 100% gross penetration of renewables. That is, the total energy production of wind/solar across all years with each layout exactly matches the total energy demand of the system over the same period; given enough storage and transmission capacity, no dispatchable generation capacity would need to be activated. To use the layouts, one need simply scale the resulting production by a factor corresponding to the desired gross penetration of the renewable resource in question.

The layouts are defined as follows. For the uniform layout, the total nameplate capacity aggregated by bus *n*∈*N* is proportional to the area aggregated by that bus, *A*_*n*_. Further, across all hours *t*∈*T*, all nodes produce energy *P*_*nt*_ equal to the total load $\sum_{m\in N,t\in T}L_{mt}$. These taken together define the capacity layout *C*_*n*_:

(9)Cnuniform=An∑mtAmPmt∑m∈N,t∈TLmt.

For the proportional layout, the installed energy at a node is proportional to both the area aggregated by that node *A*_*n*_, and the node’s average capacity factor $\sum_{t}P_{nt}/\left|T\right|$. The proportional layout can then be found as

(10)Cnproportional=An∑tPnt∑mt(AmPmt∑t′Pmt′)∑mtLmt.

Table 4 lists the total nameplate capacities found by these layouts.

To use the capacity layouts, the user should scale them to the desired degree of gross penetration. If, for example, one wishes to simulate a system with 20% gross penetration of wind in each country, one would multiply this layout by 0.20 before multiplying it with the wind energy signal. If control of the gross penetration of each country is desired, one can scale the nodes belonging to that country separately from the others, using the data provided in the network description. We finally note, that the layouts defined here have not been optimized for renewable usage, and as such may lead to a high degree of curtailment of renewable energy. Provided this is an issue in the application considered, we encourage the user to define their own renewable capacity layout.

### Code availability

The code used to generate the data set is available at^57^. All scripts have been tested working as of 20/08/2015 on machines running Ubuntu Linux 15.04, using Python version 2.7.9 with the packages Pandas version 0.15.0, Numpy 1.8.2, Scipy 0.14.0 and PyGrib 2.0.0. Certain steps in the conversion of renewable energy data may require that the user has at least 16 GB of ram. To re-generate the data set, the user would need to download the meteorological data from ECMWF^51^ and COSMO^52^, which are not provided here due to licensing. For the data from ref. 51, a MARS-access file is provided in ref. 57, which automates the download of files when updated with the user’s credentials.

The workflow when using the code is as follows (Each bullet corresponds to a folder of scripts to be executed in order):

**Network latlon** Fit positions of network buses.

**Extract ECMWF signals** Cut out first 12 h of each forecast to be used for the ECMWF signals. Also extracts a latitude-longitude grid for projection purposes.

**Interpolate ECMWF Wind** Interpolates the extracted signals to reduce the impact of the stitching of forecasts.

**Build Projection Matrix** Builds matrices to project load, wind and solar signals to the nodal domain.

**Convert Signals** Convert the extracted wind and solar fields to capacity factors. Aggregate to nodal signals.

**Convert Forecasts** Convert the forecasts for wind and solar to capacity factors. Aggregate to nodal signals.

**Make Load Maps** Project country-aggregate load signals to maps, aggregate to nodal domain.

**Build projection matrix COSMO** Build aggregation matrices for the COSMO signals.

**Convert COSMO signals** Convert COSMO signals to capacity factors, aggregate to nodal domain.

Save * CSV Save output files to.csv files.

## Data Records

All data associated with this work is available in the associated repository (Data Citation 1). The data consists of 5 parts: The transmission grid model, the generator database, the demand signal, the renewable energy forecast, the renewable energy signals and the capacity layouts, see Table 5. Each part consists of comma-separated value (.csv) files, with the first row the name of each column. Missing values are indicated by empty strings.

The transmission grid data is given in three files, one for nodes (Metadata/network_nodes.csv), one for AC transmission lines (Metadata/network_edges.csv) and one for HVDC transmission lines (Metadata/network_hvdc_links.csv). The file containing the nodes (Metadata/network_nodes.csv) is structured such that each row corresponds to a node (*n*=1,494) respectively, with columns (*n*=6) describing each node’s position and voltage (Table 6). The file for the transmission lines (Metadata/network_edges.csv) describes each transmission line (*n*=2,156), with columns (*n*=6) describing each line’s associated nodes, susceptance, number of parallel lines and limit (Table 7). A thermal limit of 0 indicates that a line has unlimited capacity. The file containing the HVDC line data (Metadata/network_hvdc_links.csv) has a single row (*n*=1) with the line’s associated nodes, limit and voltage (Table 8). We chose not to use the IEEE Common Data Format for the dataset^58^, as the inclusion of wind and solar signals and forecasts are not supported by this format.

Table 9 contains an overview of the files associated with wind, solar and load data. The files pertaining to wind and solar observations (Nodal_TS/wind_signal_ecmwf.csv, etc.) have columns corresponding to each node (*n*=1,494), with rows corresponding to the time of observation (*n*=26,304). Each observation is given as a percentage of the installed capacity. Demand data (Nodal_TS/load_signal.csv) is structured similarly, with the observations giving the hourly load in MWh. Each wind and solar forecast file (e.g., Nodal_FC/YYYYMMDDHH/wind_forecast.csv) gives the forecast for wind and solar power issued at YYYY-MM-DD HH:00:00 for the subsequent 91 h. The forecasts are given as a percentage of installed capacity.

We supply two capacity layouts (e.g., Metadata/wind_layouts_ECMWF.csv), each of which specifies the capacity in MW to install in each node to reach 100% gross renewable penetration. Each capacity layout has columns (*n*=2) corresponding to the uniform and proportional layouts, respectively, with rows (*n*=1,494) corresponding to each node. Using the capacity layout directly will result in a total energy production across all 3 years which matches the total load over all three years—i.e., given a large enough lossless storage and no thermal limits on lines, the system could be supplied fully by wind/solar power.

## Technical Validation

### Network data validation

**Figure 3:**
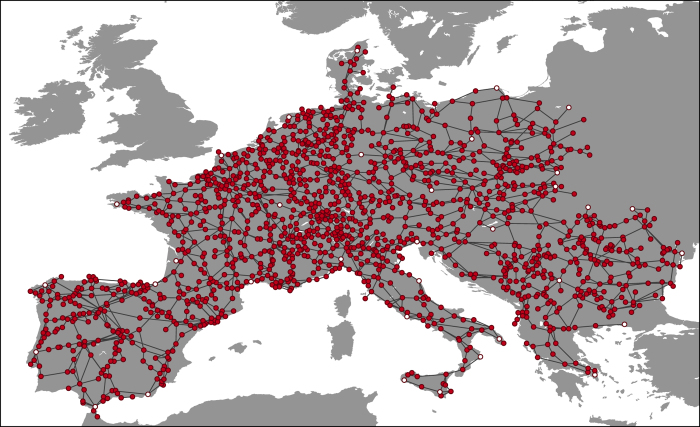
Fitted geographical coordinates for the transmission grid. White nodes are used for fitting the network.

After fitting 34 buses using a third-order polynomial, the remaining buses were transformed through the fit. The fitted network data is shown on Fig. 3, with the buses used for fitting highlighted in white. For the 34 buses which were used to calibrate the fit, the root-mean-square (RMS) error is (0.115°, 0.104°), with a maximal difference of (0.380°, 0.234°). At European latitudes, these correspond to deviations of approximately 17 km RMS and 50 km maximal. With a view to the application, the error in the fit is sufficiently small to not severely distort the hourly-averaged dynamics of the renewable energy signals. We tried out other orders of fitting polynomials, and found that a second-order polynomial was unable to properly capture the nonlinearity of the transformation, leading to a larger number of nodes positioned offshore, while a fourth-order polynomial leads to unstable behavior due to the small errors inherent in identifying buses with their associated towns. For the electrical properties of the network, we refer to the verification in ref. 11.

### Generator data validation

**Figure 4:**
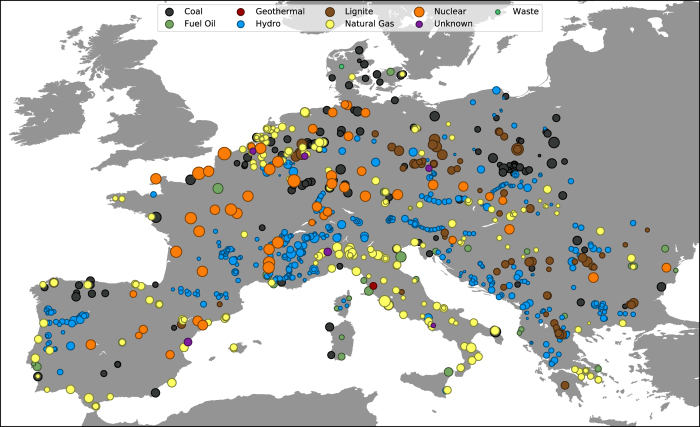
Generators in the data set. Circle area is proportional to the generation capacity of the power plant.

A map of generators in the data set is given in Fig. 4. When a generator is submitted to the underlying database^15^, administrators manually verify the data with available sources, ensuring that the information given is accurate at the time of submission. After extraction from the database, a sample of 30 data entries were manually compared with the database to ensure the conversion process was carried out correctly. A manual scan of the extracted generator data revealed a few generators with missing information on generation capacity. This information was manually filled in using data given in the GEO database^15^. Finally, some generators in the database are not connected to the mainland or are known duplicates. These were removed from the final product, and are listed in Table 10.

The cost data provided here is based on standard literature sources, and taken to depend only on the fuel type of the generator. This is standard practice in the power systems literature, as providing more accurate estimates involves detailed technical modeling which is outside the scope of the data set.

The generator database provided here covers only 78% of currently installed generation capacity in Europe, due to incompleteness of the underlying database^15^. Rather than apply ad-hoc methods for rescaling, we prefer to leave the issue of how to compensate for the lack of coverage up to the user.

### Wind signal interpolation

For the ECMWF-derived wind signal, the interpolation method given by equation (5) leaves the number of hours of interpolation undefined. In order to quantify this, we look at the spatially averaged 1-hour root-mean-square change in the wind speed fields:

(11)ΔW(t)=〈(W(x,t+1)−W(x,t))x2〉x,

which gives a measure of the typical wind speed change. Here, *W*(*x*, *t*) is the raw wind speed at grid position *x* at time *t*. We expect this quantity to change slowly over time, as it essentially measures changes in the energy available in the near-surface wind layer.

Indeed, Δ*W*(*t*)=0.726±0.12 m/s on average for times away from the seam between forecasts, while Δ*W*(*t*)=1.06±0.13 m/s across the forecast seam. After interpolating over 1 hour every 12 h, Δ*W*(*t*) shrinks to 0.749±0.11 m/s, within the range of typical behavior. By further examining the differential of Δ*W*(*t*), and by plotting the interpolated wind fields, interpolation across 1 hour every 12 h was found to give the best compromise between smoothing out the seam and introducing the smallest change in the affected fields.

### Comparison of solar and wind signals to real-world signals

We aim here at analysing how our datasets composed of forecasts and observation signals reproduce some of the salient features from a real large scale power system. This is not to be seen as a strict comparison with reality, as the data background which would be required for such a comparison is not available in practice. This lack of availability is one of the very reason which motivated the generation of this dataset in the first place.

**Figure 5:**
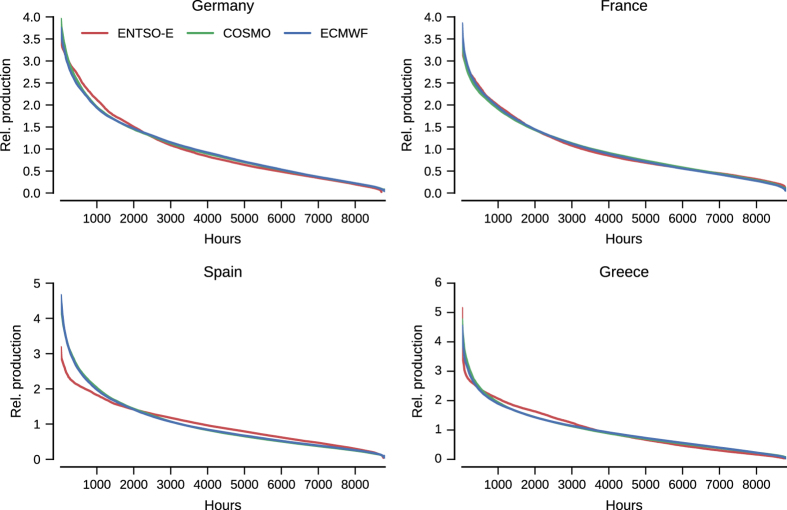
Wind duration curves versus ENTSO-E data from 2015, aggregated over the indicated country. All curves are normalized to have mean 1.

**Figure 6:**
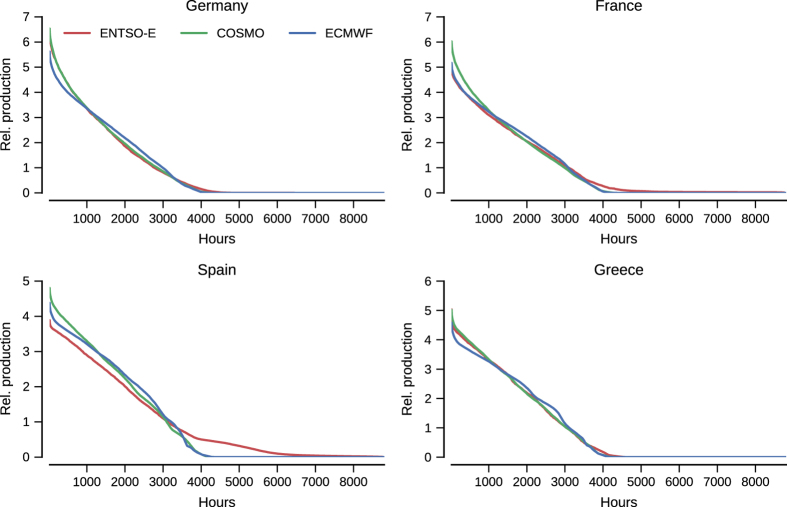
Solar duration curves versus ENTSO-E data from 2015, aggregated over the indicated country. All curves are normalized to have mean 1.

Consequently here, we compare our data with signals at a more aggregated level. For that purpose, the reference data for comparison is hourly generation data for wind and solar power in 2015 extracted from the ENTSO-E transparency portal^45^. To be consistent, a country-aggregated signal is obtained from our dataset, using the ‘proportional’ capacity layout. As our aim is not to replicate real-world data exactly, but merely to show that the trends are largely captured by the data, we focus on duration curves of production. In addition, since these signals differ in overall scale due to differences in the installed capacity, we scale them to have mean 1. Resulting sample duraction curves for the case of wind and solar power are depicted in Figs 5 and 6, respectively.

In both cases, i.e., for wind and solar power, the agreement of duration curves between generated datasets and aggregated data from ENTSO-E displays a reasonable qualitative agreement. This is especially true, in view of the simple assumptions used as a basis, as well as the limited knowledge of all renewable power generation capacities over Europe.

Even so, for some countries, e.g., Spain here for the wind data, the generated dataset tend to have more frequent low production hours than in the reality. This could be explained by difference in capacity layouts, or fundamentally by a bias in the low wind speed range of the input meteorological data. In parallel, for the solar case, Spain is also an example of higher mismatch between generated and ENTSO-E data. There, Spain’s solar signal includes power generation of concentrating solar power units, whose production stretches into the night as power production can be delayed. This inflates the mean, leading to a lower curve overall, and causes the bump between 4,000 and 6,000 h. This indicates, that a future version of the dataset should model concentrating solar power to properly model the Spanish generation mix.

It should be noted that most of the information content in the renewable energy signals originate from the meteorological data used, which are nonlinearly converted to power production. As these forecasts and re-analysis data sets are generally validated in a thorough manner by the meteorological community, they can represent variations over areas smaller than the country scales at which this comparison takes place. Thus, one should expect the correspondance shown here to project to smaller length scales, bounded by the error made in the power conversion choice.

### Forecast verification

The dataset contains three sets of renewable production data: A point forecast derived from ECMWF weather data (ECMWF-Forecast), a continuous signal derived from COSMO-REA6 weather reanalysis data (COSMO-Signal), and a second continuous signal stitched together from ECMWF weather data (ECMWF-Signal). While ECMWF-Forecast and ECMWF-Signal have model consistency, ECMWF-Forecast and COSMO-Signal lack it. One should thus expect ECMWF-Forecast to be a better predictor for ECMWF-Signal than for COSMO-Signal.

**Figure 7:**
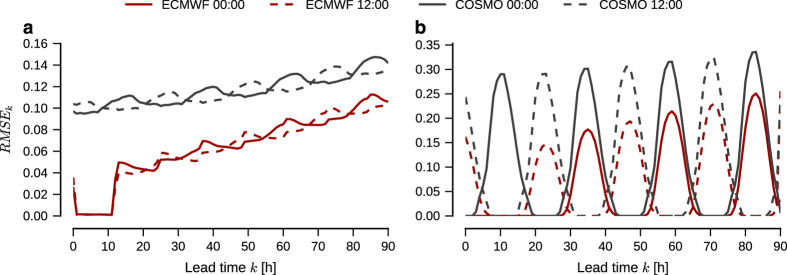
Root-mean-square errors for wind and solar forecasts. Plots of (12) for (**a**) wind and (**b**) solar forecasts against ECMWF (red) and COSMO (black) signals, plotted for all lead times. Fully drawn curves are for forecasts issued at midnight, dashed curves for ones issued at noon.

To evaluate the forecasts, we calculate the root-mean-square error

(12)RMSEk=1NT∑nt(Pˆntk−Pnt)2,

where ${\hat{P}}_{ntk}$ is the point forecast of capacity factor for node *n* at time *t*, given *k* hours in advance, and *P*_*nt*_ is the corresponding observed production. The resulting curves are plotted on Fig. 7 for both signals, split depending on whether the forecast was issued at noon or midnight.

Some comments on the curves are in order. First, note that the wind forecasts on Fig. 7 graph (a) for the ECMWF signal display no forecast errors for k=1,…,11h. The same is true for solar on graph (b) in the same figure at k=1,…,23h for the forecast issued at midnight. This is not an error, but due to the construction of the ECMWF signal from forecasts. The signal uses the first 12 h of each wind forecast, and the first 24 h of each solar forecast issued at midnight, where it will naturally agree exactly with the forecast. Second, the forecast error for the COSMO signals are significantly higher than for the ECMWF signals. This is due to inherent biases in the underlying signals due to differences in their derivation, spatial resolution and modeling methods. No attempt has been made for this version of the data set to correct for these biases; we leave this as a task for future research. Further, as the ECMWF data is naturally consistent with itself, it is natural that the forecast is closer to the ECMWF signal than the COSMO signal.

Our choice for an RMSE criterion is in line with current practice in the renewable energy forecasting community, which sees it as a lead error measure to report. Additional criteria., e.g., bias and Mean Absolute Error (MAE) could also be reported, while a finer analysis could be based on diagnostic tools and error distributions.

### Evaluation of full dataset

We have run simple linear economic dispatch models using the dataset and found that no load shedding is necessary for any level of renewables. These economic dispatch models employed no unit commitment constraints or other constraints coupling across timesteps. Thermal limits on the full transmission grid was taken into account, with the coupling of flows to bus phasors represented in the DC-approximation. Further, load shedding was modeled as a high-cost generator at each bus, limited by the load in that bus.

These results indicate, that the generation capacity detailed in the data is sufficient to supply load in the linearized case, indicating that the lack of full coverage of the generator database is not critical to applicable studies.

In view of the multiple and diverse foreseen applications of the dataset, we consider it outside the scope of this manuscript to attempt a full evaluation of the dataset’s efficacy for various applications. Instead, we encourage users of the dataset to identify caveats and limitations for their specific use case, and to provide feedback in order for the dataset to evolve and improve as a community initiative.

## Usage Notes

The dataset is available from Zenodo as indicated in Data Citation 1, with the source code available from ref. 57.

Most aspects related to use of the data set are self-explanatory, but we would like to insist on the following two points. First, network admittances are given per-unit for the connection as a whole; it is not necessary to correct these for voltage levels, length of the line or number of parallel lines. Second, renewable production data is given as capacity factors, which necessitates the use of a capacity layout. The capacity layouts included in the data set are scaled such that if they are used directly, the total production of wind or solar matches total load across all 3 years, i.e., for a gross penetration of 100%. These capacity layouts do not correspond to the currently installed capacity.

For a user looking to re-generate the dataset, Table 11 sketches the data needed. An extensive list of data sources required and the processing thereof is given in the Github repository^57^.

## Additional Information

**How to cite this article:** Jensen, T. V. & Pinson, P. RE-Europe, a large-scale dataset for modeling a highly renewable European electricity system. *Sci. Data* 4:170175 doi: 10.1038/sdata.2017.175 (2017).

**Publisher’s note:** Springer Nature remains neutral with regard to jurisdictional claims in published maps and institutional affiliations.

## Supplementary Material



## Figures and Tables

**Table 1:** Dataset components.

**Dataset components**		
Network	1,494 buses	2,156 lines
Generators	969 units	Technical & cost data
Demand	3 years	Hourly per bus
Wind & solar production	3 years	Hourly per bus
Wind & solar forecast	91 h at 00:00 & 12:00		

**Table 2:** Estimated costs for fuel only.

**Fuel type**	**Fuel cost**	**Heat value [GJ/t]**	**Thermal efficiency [%]**	**Marginal cost of fuel [$/MWh]**
Coal	60$/t	24.0	40.5	22.2
Lignite	20$/t	24.0	40.5	7.4
Natural Gas	3.76 $/GJ	—	54	25.1
Fuel Oil	563$/t	40.7	54	92.2

**Table 3:** Generator parameters for unit commitment problems.

**Fuel type**	**Min. up/downtime**	**Cycling cost [$/MW]**	**Min. online capacity [%]**	
	**Up [h]**	**Down [h]**		
Biomass/waste	8	8	65	30
Coal	8 (4–48)	8 (8–24)	65 (55–78)	30
Fuel Oil	2 (1–10)	4 (1–12)	55	40
Geothermal	0	0	55	15
Hydro	0	0	4.3 (2.1–8.6)	15
Lignite	8	8	65	30
Natural Gas	2	4	55 (32–93)	40
Nuclear	24	24	300	20 (20–50)
Undefined	8	8	65	40
Quantities in parens indicates the range given in the applicable source.				

**Table 4:** Total nameplate capacities of layouts at 100% gross penetration.

	**ECMWF**		**COSMO**	
**Capacity [GW]**	**Uniform**	**Proportional**	**Uniform**	**Proportional**
Solar	1,048	1,032	1,284	1,259
Wind	1,734	1,207	1,554	1,186

**Table 5:** Number of files and sizes in full data set.

**Field type**	**No. of files**	**Total size**	**Notes**
Network data	3	175 KB	
Generator data	1	141 KB	
Demand signal	1	336 MB	
Wind and solar signals	4	1.07 GB	2x Wind, 2x Solar
Wind and solar forecasts	3,834	1.5 GB	Forecasts issued at h and 12 h
Wind and solar capacity layouts	4	136 KB	

**Table 6:** Summary of columns in node data.

**Col.**	**Col. name**	**Format**	**Units**	**Range**	**Description**
1	ID	int	—	1–1,514	Node ID
2	name	string	—	—	Name of bus in^11^
3	country	string	—	—	3-letter country ISO code
4	voltage	float	kV	110–380	Bus voltage
5	latitude	float	°N	35.5–57.3	Latitude (WGS84)
6	longitude	float	°E	−9.24–29.0	Longitude (WGS84)

**Table 7:** Summary of columns in AC line data.

**Col.**	**Col. name**	**Format**	**Units**	**Range**	**Description**
1	fromNode	int	—	1–1,508	Origin node ID
2	toNode	int	—	2–1,514	Destination node ID
3	X	float	p.u.	10^−5^–0.577	Line reactance (10^−5^=no data)
4	Y	float	p.u.	1.73–10^5^	Line susceptance (=1/X)
5	numLines	int	—	1–4	Number of parallel lines
6	limit	float	MW	0–15,000	Thermal flow limit (0=unlimited)
7	length	float	km	8.5–256	Line length from bus positions

**Table 8:** Summary of columns in DC line data.

**Col.**	**Col. name**	**Format**	**Units**	**Range**	**Description**
1	ID	string	—	—	DC line ID
2	fromNode	int	—	1,514	Origin node ID
3	toNode	int	—	1,513	Destination node ID
4	limit	float	MW	500	Thermal flow limit (0=unlimited)
5	length	float	km	313	Line length from bus positions

**Table 9:** Structure of load, wind and solar signals, wind and solar forecasts and capacity layouts.

**Signal type**	**Col. header**	**First column**	**Remaining columns**
Wind/solar signal	Node ID	Time	Capacity factor [% of max cap.]
Wind/solar forecast	—"—	—"—	—"—
Load signal	—"—	—"—	Hourly demand [MWh]
Wind/solar layout	Layout type	NodeID	Assigned capacity at 100% gross penetration [MW]
Time is given in as YYYY-MM-DD hh:mm:ss.			

**Table 10:** Generators removed from extracted data.

**ID**	**Note**
2,402	Duplicate of 39,749
2,638	Duplicate of 39,746
42,778	Crete, Greece
42,779	Chios, Greece
43,804	Canary Islands, Spain
43,815	Canary Islands, Spain

**Table 11:** List of fields and files needed to re-derive the data set.

**Source**		**Fields/Files**	**Note**
ECMWF	^51^	P165, P166, P167, P169, P243, P246, P247 lafYYYYMMDDHH0000; wind-u (×6), wind-v (×6),	MARS access file available at^57^
COSMO-REA6	^52^	Temp 2 m, Ibeam, Idiffuse, Iground	Inventory example available at^57^
Global Energy Observatory	^15^	GEO_PP_[Type] _[Country]_2000-2009.kml	All applicable types for covered countries
ENTSO-E	^45^	[Country]_[Year].xls	Country packages

## References

[d1] Zenodo Jensen T. de Sevin H. Greiner M. Pinson P. 2017 https://doi.org/10.5281/zenodo.999150

